# KRAS, TP53, CDKN2A, SMAD4, BRCA1, and BRCA2 Mutations in Pancreatic Cancer

**DOI:** 10.3390/cancers9050042

**Published:** 2017-04-28

**Authors:** Jonas Cicenas, Kotryna Kvederaviciute, Ingrida Meskinyte, Edita Meskinyte-Kausiliene, Aiste Skeberdyte, Jonas Cicenas

**Affiliations:** 1 Vetsuisse Faculty, Institute of Animal Pathology, University of Bern, Länggassstrasse 122, 3012 Bern, Switzerland; 2 MAP Kinase Resource, Bioinformatics, Melchiorstrasse 9, 3027 Bern, Switzerland; 3 Proteomics Centre, Institute of Biochemistry, Vilnius University, Sauletekio al. 7, LT-10257 Vilnius, Lithuania; 4 Faculty of Mathematics and Informatics, Vilnius University, Naugarduko st. 24, LT-03225 Vilnius, Lithuania; k.kvederaviciute@mapkinases.eu; 5 Ministry of Education and Science, A. Volano g. 2, LT-01516 Vilnius, Lithuania; ingrida.meskinyte@gmail.com; 6 Energy and Biotechnology Engineering Institute, Aleksandro Stulginskio University, Studentų g. 11, LT-53361 Akademija, Lithuania; edita.meskinyte@gmail.com; 7 Division of Human Genome Research Centre, Institute of Biosciences, Life Sciences Center, Vilnius University, Sauletekio al. 7, LT-10257 Vilnius, Lithuania; aiste.skeberdyte@gmail.com; 8 International School of Law and Business, Laisvss pr. 58, LT-05120 Vilnius, Lithuania; jo.cicenas@gmail.com

**Keywords:** KRAS, TP53, CDKN2A, SMAD4, BRCA1, BRCA2, mutation, pancreatic cancer, genetic variant

## Abstract

Pancreatic cancer is a disease that has a very high fatality rate and one of the highest mortality ratios among all major cancers, remaining the fourth leading cause of cancer-related deaths in developed countries. The major treatment of pancreatic cancer is surgery; however, only 15–20% of patients are candidates for it at the diagnosis of disease. On the other hand, survival in patients, who undergo surgery, is less than 30%. In most cancers, genome stability is disturbed and pancreatic cancer is not the exception. Approximately 97% of pancreatic cancers have gene derangements, defined by point mutations, amplifications, deletions, translocations, and inversions. This review describes the most frequent genetic alterations found in pancreatic cancer.

## 1. Introduction

As opposed to steady or diminishing rates of most solid tumors, pancreatic cancer remains one of the most deadly cancers. More than 43,000 patients are expected to die of pancreatic cancer in the USA and 91,500 in the European Union in 2017 [1,2]. There are also some alarming predictions that pancreatic cancer might soon become the second highest cause of cancer-related death in the USA and the third highest in the EU. In addition, virtually the only possible treatment of pancreatic cancer is surgery, but less than 20% of patients have operable tumors, and less than 30% of those patients survive surgery [3,4]. Moreover, diagnosis of pancreatic cancer is quite challenging because histological tests require invasive approaches, and high amounts of stromal cells in the tumor cause biopsies to be insufficient for correct diagnosis [4,5]. Given all of this, pancreatic cancer is one of the most investigated cancers both in basic and clinical science setups. 

**Figure 1:**
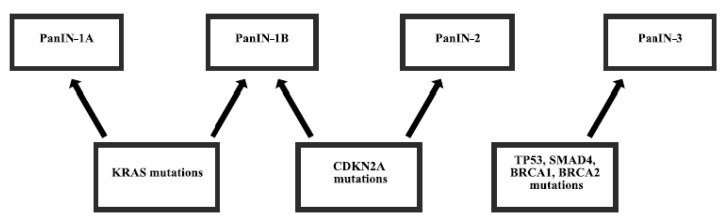
Mutations in the carcinogenesis of pancreatic adenocarcinoma. Pancreatic intraepithelial neoplasia (PanIN) grading.

Most pancreatic cancers (97%) have gene alterations, such as amplifications, deletions, translocations, inversions, frameshifts, and substitutions. Disruptions of several genes in pancreatic cancer are almost universal and are present in 70%–98% of patients [5,6,7]. Mutations of other genes are less frequent [8] and some of them are derived from germline inheritance [9,10] (Figure 1). The mutation of genes can either enhance the function of protein, sometimes making it even constitutively active or diminish the function, sometimes abolishing it completely. Subsequently, the changes in protein functions can cause uncontrolled proliferation, motility, and adhesion of cells, protection from apoptosis or autophagy, DNA repair problems, microsatellite instability, and other processes, which lead to the development, growth, and spread of cancer.

## 2. Gene Mutations in Pancreatic Cancer

**Figure 2:**
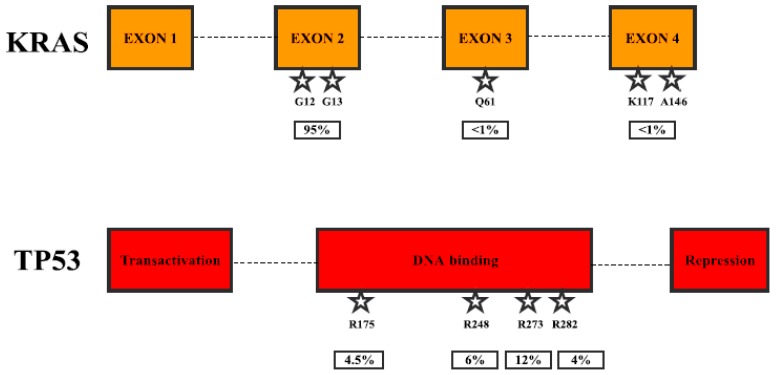
Most frequent mutations in pancreatic cancer. KRAS mutations by the exons and TP53 mutations by protein domain.

*KRAS* (also known as K-Ras 2, Ki-Ras, c-K-ras, or c-Ki-ras) is a small GTPase (21 kDa), which binds guanosine triphosphate and diphosphate nucleotides. It is activated when bound to GTP and deactivated when bound to GDP. Activated KRAS binds and activates RAF family kinases, RAF1, BRAF, and ARAF [11]. Activated RAFs phosphorylate and activate MEK1 and MEK2 kinases, which in turn phosphorylate and activate ERK1 and ERK2 kinases. ERKs phosphorylate various cytosolic and nuclear proteins, such as transcription factors ELK1 and c-JUN, leading to cell proliferation [12]. Due to this, mutations, which cause constitutive activation of KRAS, lead to uncontrolled proliferation and other processes causing cancer development and spreading. KRAS can also regulate other signaling pathways, such as PI3K-AKT, PLC-PKC, and RAL, which are also known to be involved in cancer progression [13]. KRAS is mutated in more than 20% of human cancers, mostly in pancreatic (more than 90%), colorectal and lung cancers [13] as well as leukemias [14] (Table 1). Mutations of the codons G12, G13, or Q61 are usually associated with constitutively active KRAS, and recurrent mutations in K117 and A146 seem to be additional hotspots (Figure 2). Ninety-five percent of pancreatic cancers carry activating mutations in KRAS and modifications in G12 account for 99% of all mutations (G12D—50%) [15]. On the other hand, G13 mutations are much rarer than in some other cancers (e.g., colorectal 17%). Clinical studies have shown that mutations of KRAS could be used as a significant prognostic biomarker, as well as a tool for therapy prediction. In a study that examined 136 pancreatic adenocarcinoma patients, 71 (52%) patients harbored point mutation in G12 (70) and Q61 (1) [16]. Patients with mutations showed a worse response to first-line gemcitabine-based chemotherapy (11.3%) than those with wild-type KRAS (26.2%) and poorer survival (*p* = 0.001). In addition, survival benefit was observed in the subgroup of patients treated with gemcitabine/erlotinib combination (*p* = 0.002), but no survival difference was observed in the subgroup of patients treated only with gemcitabine (*p* = 0.121). Another study in 173 patients, 121 (70%) of which had G12 mutations, showed no association with response to erlotinib alone (*p* = 0.4), but KRAS wildtype patients had an improved overall survival (*p* = 0.005) [17]. Phase I/II study using siRNA against G12D in combination with chemotherapy was performed in 15 locally advanced pancreatic cancer patients [18]. None of the 12 patients analyzed later showed tumor progression, 10 had stable disease, 2 displayed partial response, and median overall survival was 15.12 months. The study using a targeted deep sequencing assay detected KRAS mutations in 96/100 (96%) patients. The patients were then separated into two groups: one having 0–2 mutations, and the other having 3. The presence of 3 mutations was prognostic for a better overall survival (*p* = 0.004) [19]. Interestingly, activating mutations in kinase downstream of KRAS signaling, BRAF (V599E), were found in 3/9 (33%) tumors, lacking KRAS mutations, but known to have microsatellite instability [20].

*TP53* (also known as p53 or antigen NY-CO-13) is the tumor suppressor, which transcriptionally activates target genes in response to cellular stress such as oxidative stress or DNA damage and thus induces growth arrest or apoptosis [21]. It also increases cyclin-dependent kinase inhibitor CDKN1A expression, thus stopping cell cycle progression [22]. TP53 is one of the most frequently mutated genes in all cancers and is mutated in 70% of pancreatic cancers [23], mostly resulting in the loss of DNA binding ability and thus subsequently gene transcription activation [24] (Figure 2, Table 1). Some clinical evidence suggests that TP53 could be used as biomarker for prognosis and therapy prediction. A study in 57 pancreatic ductal adenocarcinoma patients assessed TP53 mutations as well as mRNA expression [25]. It was observed that patients with low TP53 mRNA expression were associated with worse prognosis (*p* = 0.032), and the results were more significant in patients with TP53 wild-type genes (*p* = 0.021). One quite promising study was published recently, stating that mutated TP53 caused poor prognosis to pancreatectomy through upregulation of PTRF (cavin-1) in patients with preoperative serum [26], but the publication was retracted due to the use of wrong antibodies in the study [27]. Nevertheless, the result seems to be interesting, and it is hopeful that the study will be repeated using suitable techniques. A study in pancreatic cancer patient tumors, of which 4/50 (8%) had a complete loss of TP53 expression, 20/50 (40%) showed regular expression, and 26/50 (52%) patients showed overexpression, showed a significant improvement in progression-free survival (*p* = 0.02) for patients with regular expression compared to complete loss [28]. The study using a targeted deep sequencing assay detected TP53 mutations in 13/100 (13%) patients, and the presence of 0–2 vs. 3 mutations was prognostic for better overall survival (*p* = 0.004) [19].

*CDKN2A* (also known as p16-INK4a, MTS-1, or CDK4I) is the tumor suppressor, which regulates cell cycle progression by inhibiting cyclinD-CDK4 and cyclinD-CDK6 complexes responsible for initiating the G1/S phase transition. This protein encoded by the CDKN2A gene, but is unrelated to another tumor suppressor ARF, encoded by the same gene. Inherited modifications in CDKN2A cause familial atypical multiple mole melanoma and an increased risk of pancreatic cancer [29,30]. It is one of the most frequently altered genes in cancer, and the incidence of mutations in sporadic pancreatic cancer is impressive, with inactivation occurring in 98% of cases [31] (Table 1). CDKN2A gene disruption happens by different types of mutations, such as the loss of heterozygosity, homozygous deletion, or promoter silencing. There is some clinical evidence for the use of CDKN2A mutations as prognostic and predictive biomarker. The study performed in 88 pancreatic cancer patients, 69 of whom underwent pancreaticoduodenectomy and 19 did not undergo resection [32]. In 3’UTR C580T, the mutation rate was different between patients that had pancreaticoduodenectomy and patients that did not. In patients with surgery, 56 (81%) had the CC genotype, 13 (19%) had the CT genotype, and none had the TT genotype, and of the patients without surgery, 12 (63%) had the CC genotype, 5 (26%) had the CT genotype, and 2 (11%) had the TT genotype. There was significant disease-free survival between CC and CT/TT genotypes (*p* = 0.039). In another study, 5/120 (4%) of pancreatic cancer patients had CDKN2A mutations, namely G101W, E27X, and L65P [33]. Three of those patients had melanoma-pancreatic cancer kindred, suggesting that cancers were familial. Since only about 10% of pancreatic cancers are part of all familial cancers, the results showing substantially high mutation rates suggested that detected CDKN2A mutations are prevalent in the region where analysis was performed. Fluorescence in situ hybridization study showed CDKN2A deletion in 16/32 (50%) patients and overall survival was shorter in patients with gene deletion (*p* = 0.002) [34]. The study using a targeted deep sequencing assay detected CDKN2A mutations in 42/100 (42%) patients, and the presence of 0–2 vs. 3 mutations was prognostic for better overall survival (*p* = 0.004) [19].

*SMAD4* (also known as DPC4 or MADH4) is tumor suppressor protein, which translocates to the nucleus as heterotrimeric SMAD2/SMAD3-SMAD4 complex after TGFβ family receptors activation, where it activates the expression of genes and causes growth inhibition [35,36]. Mutations of SMAD4 occur in around 50% of pancreatic cancers, mostly leading to the loss of protein activated [37] (Table 1). Approximately 30% of mutations occur by homozygous deletion. Clinical research results show that SMAD4 inactivation by mutations could be used as a prognostic biomarker in pancreatic cancers. The study in 90 pancreatic cancer patients showed that SMAD4 mutations were present in 17/90 patients (19%) and gene mutation status was significantly associated with overall survival (*p* = 0.006) [38]. In another study, SMAD4 gene was inactivated in 8/25 (32%), 3/25 (12%) by homozygous deletion, and 5/25 (20%) by mutations in MH2 domain [39]. The study using a targeted deep sequencing assay detected SMAD4 mutations in 7/100 (7%) patients, and the presence of 0–2 vs. 3 mutations was prognostic for better overall survival (*p* = 0.004) [19].

*BRCA1* (also known as RNF53) is a tumor suppressor, which acts as E3 ubiquitin-protein ligase that mediates the formation of Lys-6-linked polyubiquitin chains [40]. It is regulating cellular responses to DNA damage and thus is very important in DNA repair and G2/M cell cycle progression [41]. The gene is frequently mutated in quite a few familial cancers: it accounts for 45% of families with a high incidence of breast cancer and 80% of families with an elevated frequency of both breast and ovarian cancer [42]. Most of the BRCA1 mutations (88%) result in protein truncation and thus result in the loss of function of the protein and subsequently contribute to tumorigenesis. On the other hand, sporadic mutations of this gene is quite rare. Mutations of BRCA1 occur in approximately 6.6% of pancreatic patients and could be associated with familial pancreatic cancer risk [43] (Table 1). There is no direct clinical evidence that BRCA1 could be used as predictive biomarker, but a preclinical study using patient-derived xenografts showed that BRCA1 and BRCA2 mutations sensitize tumors to cisplatin chemotherapy [44].

*BRCA2* (also known as FANCD1) is involved in double-strand break repair during the S phase of the cell cycle, by activating RAD51 recombinase. It is also involved in cytokinesis, centrosome duplication, and cell death [45]. Mutated and thus frequently inactivated BRCA2 cause an increased risk of developing breast, ovarian, prostate, stomach, and pancreatic familial cancers [46,47]. Mutations of BRCA2 are found in approximately 7.3% of familial pancreatic cancer patients and show the increased risk by up to 20-fold [9] (Table 1). However, in the case of sporadic cancer, very few cases of somatic mutations of BRCA2 are reported. A number of clinical studies were performed to estimate BRCA2 as potential prognostic and predictive biomarker for pancreatic cancer. The two-stage association study on pancreatic cancer that included 981 cases and 1991 controls in the first stage and 2603 cases and 2877 controls in a second stage showed that c.*532A>G variant located in the 3′-UTR is significantly associated with sporadic pancreatic cancer (*p* < 0.0001) [48]. A patient carrying rare BRCA2 1153insertionT mutation was unresponsive to gemcitabine therapy, but complete remission was reached, when cisplatin was added in combination with gemcitabine [49]. Another case of a pancreatic cancer patient with a 6174delT BRCA2 mutation showed prolonged survival after docetaxel, capecitabine, and gemcitibine combination followed by single agent irinotecan, despite prognostically unfavorable disease [50]. There was also a case of a patient with metastatic pancreatic cancer responding to a combination of mitomycin C and capecitabine [51]. All these cases combined show that BRCA2 mutations can be used as predictive biomarkers for increased sensitivity of pancreatic tumors to DNA-intercalating agents.

## 3. Conclusions

Most cancers, including pancreatic, are very genetically complex diseases. Many cancers develop because of gene mutations, which either render proteins in cells defective or totally delete them. Therefore, cancer research is striving towards the detection of as many genetic alterations in important genes as possible. New generation sequencing is an incredible technique, which helps to classify and systematize the entire range and characteristics of somatic and germline alterations [52]. It is now reaching a point where such a technique is going to be used by many laboratories for routine diagnostics, prognostics, and therapy prediction. There are also a growing number of companies that specialize in bioinformatics applications for clinical scientists to investigate data sets with hundreds of patient gene mutations and related clinical parameters. Up to now, gene mutation studies have discovered approximately 140 genes that can promote tumorigenesis when mutated. Normally, a tumor contains 2–8 of these “driver” mutations and the rest of them are passengers with no particular growth advantage for the tumor. It is important to reach a point at which our knowledge of the genes mentioned in this review will be sufficient to evaluate pancreatic cancer morbidity, mortality, and therapeutic approaches.

## Figures and Tables

**Table 1:** Gene mutation frequency in pancreatic cancer.

Gene	Frequency	Reference
KRAS	70%–95%	[15,16]
TP53	20%–76%	[23,24]
CDKN2A	49%–98%	[31,32]
SMAD4	19%–50%	[37,38]
BRCA1	6.6%–14%	[43,44]
BRCA2	3.6%–7.5%	[9,10,43,44]
